# The Loss of *Gnai2* and *Gnai3* in B Cells Eliminates B Lymphocyte Compartments and Leads to a Hyper-IgM Like Syndrome

**DOI:** 10.1371/journal.pone.0072596

**Published:** 2013-08-19

**Authors:** Il-Young Hwang, Chung Park, Thuyvi Luong, Kathleen A. Harrison, Lutz Birnbaumer, John H. Kehrl

**Affiliations:** 1 B Cell Molecular Immunology Section, Laboratory of Immunoregulation, National Institute of Allergy and Infectious Diseases, National Institutes of Health, Bethesda, Maryland, United States of America; 2 Laboratory of Neurobiology, National Institute of Environmental Health Sciences, National Institutes of Health/Department of Health and Human Services, Durham, North Carolina, United States of America; University Paris Sud, France

## Abstract

B lymphocytes are compartmentalized within lymphoid organs. The organization of these compartments depends upon signaling initiated by G-protein linked chemoattractant receptors. To address the importance of the G-proteins Gα_i2_ and Gα_i3_ in chemoattractant signaling we created mice lacking both proteins in their B lymphocytes. While bone marrow B cell development and egress is grossly intact; mucosal sites, splenic marginal zones, and lymph nodes essentially lack B cells. There is a partial block in splenic follicular B cell development and a 50-60% reduction in splenic B cells, yet normal numbers of splenic T cells. The absence of Gα_i2_ and Gα_i3_ in B cells profoundly disturbs the architecture of lymphoid organs with loss of B cell compartments in the spleen, thymus, lymph nodes, and gastrointestinal tract. This results in a severe disruption of B cell function and a hyper-IgM like syndrome. Beyond the pro-B cell stage, B cells are refractory to chemokine stimulation, and splenic B cells are poorly responsive to antigen receptor engagement. Gα_i2_ and Gα_i3_ are therefore critical for B cell chemoattractant receptor signaling and for normal B cell function. These mice provide a worst case scenario of the consequences of losing chemoattractant receptor signaling in B cells.

## Introduction

*Gnai1*, *Gnai2*, and *Gnai3* encode members of the “inhibitory class” of heterotrimeric G-proteins so named based on their ability to inhibit adenylyl cyclase activity [1]. Targeted loss-of-function mutations of *Gnai1*, *Gnai2*, and *Gnai3* have been generated in mice revealing redundancy as well as tissue specific functions for *G*α_i1_, Gα_i2_, and Gα_i3_ that partially depend upon the genetic background of the mice [2,3,4,5,6]. Gnai2^-/-^ mice have a reduced relative abundance of splenic marginal zone and T2 transitional B lymphocytes, reduced peritoneal B-1a B cells, and increased splenic follicular and peritoneal Β-1b B cells [7]. These phenotypes arise as a consequence of a lymphocyte intrinsic defect as Gnai2^-/-^ bone marrow reconstituted RAG2^-/-^ mice have a similar B cell phenotype [7]. Providing some insight into these phenotypes, B cells from the Gα_i2_ deficient mice have diminished responses to chemoattractants [8]. Furthermore, adoptively transferred Gnai2^-/-^ B cells adhere poorly to high endothelial venules (HEVs) and enter inefficiently into lymph nodes. Those B cells that do enter tend to accumulate around the HEVs rather than normally transiting into the CXCL13-rich follicles [9]. In contrast, B cells from Gnai3^-/-^ mice exhibit few if any defects in their chemoattractant responses [10]. Since lymphocytes express little Gα_i1,_ it is likely irrelevant to B lymphocyte trafficking.

The analysis of chemoattractant receptor targeted mice has revealed roles for CXCR4, CXCR5, CCR6, CCR7, CCR9, CCR10, S1PR1, CNR2, and GPR183 (EBI2) in B lymphocyte function. CXCR4, S1PR1, and CNR2 regulate the retention and egress of B lymphocyte in the bone marrow [11,12,13]. CCR7 and CXCR5 mediate the entrance of B cells into most lymphoid organs and assist in the positioning of B cells at different sites [14,15,16,17]. CXCR4 and GPR183 also function in the positioning of B cells in lymphoid organs [18,19,20]. Besides its role in bone marrow egress S1PR1 assists the egress of B cells from lymph nodes and Peyer’s patches [21,22]. CCR9/10 help target plasma cells to the lamina propria of the gut [23,24,25]. Finally, CCR6 assists in the recruitment of B cells to the skin, inflammatory sites, and isolated lymphoid follicles [26,27]. Based on their sensitivity to pertussis toxin all these receptors likely use Gα_i_ containing heterotrimeric G-proteins to link to downstream effectors. Pertussis toxin ADP ribosylates a cysteine residue near the C-termini of Gα_i1-3_, which inhibits G-protein coupled receptor (GPCR) triggered nucleotide exchange [28]. To test the explicit role of Gα_i2_ in B lymphocytes we created mice in which a portion of the coding region of *Gnai2* is flanked by loxP sites (*Gnai2* ^fl/fl^), recombination signals for the *cre* recombinase. We crossed these mice to *mb1-cre* knock-in (KI) mice [29], thereby deleting a portion of the *Gnai2* coding sequence in B cells and causing a loss of Gα_i2_ in those cells. To determine the functional importance of Gα_i3_ in B lymphocytes lacking Gα_i2_ we crossed the *Gnai2* ^fl/fl^ *mb1-cre* mice to the Gnai3^-/-^ mice. We compared B lymphocytes from *Gnai2* ^fl/fl^, *Gnai2* ^fl/fl^ *mb1-cre*, *Gnai2* ^fl/fl^Gnai3^-/-^ (referred to as Gnai3^-/-^), and Gnai3^-/-^/*Gnai2* ^fl/fl^ *mb1-cre* (referred to as DKO) mice. This analysis provides insights into the importance of Gα_i2_ and Gα_i3_ for B cell responses to chemoattractants and B cell function.

## Materials and Methods

### Animals

C57BL/6, and B6.SJL-Ptprc^a^ Pepc^b^/BoyJ mice were obtained from Jackson Laboratory. *Gnai2* ^fl/fl^ mice and Gnai3^-/-^ mice on a mixed background have been described [6]. Both strains were each backcrossed 11 times on to C57BL/6. The C57/BL6 *mb1-cre* mice were kindly provided by Dr. Michael Reth (University of Freiburg, Germany). For bone marrow reconstitution, seven weeks old B6.SJL-Ptprc^a^ Pepc^b^/BoyJ (CD45.1) mice were irradiated twice with 550 rads for total of 1100 rads and received bone marrow from *Gnai2* ^*fl/fl*^ C57BL/6 CD45.2 mice (control) or from DKO C57BL/6 CD45.2 mice. The engraftment was monitored by sampling the blood 28 days later. The mice were used 6-8 weeks after reconstitution. All mice were used in this study were 6-14 weeks of age. Mice were housed under specific-pathogen-free conditions. All the animal experiments and protocols used in the study were approved by the NIAID Animal Care and Use Committee (ACUC) at the National Institutes of Health.

### Cells

Splenic B cells were isolated by negative depletion using biotinylated antibodies to CD4, CD8, Gr-1 (Ly-6C and Ly 6G), and CD11c and Dynabeads M-280 Streptavidin (Invitrogen) as previously described [22]. The B cell purity was greater than 95%. When needed B cells were cultured in RPMI 1640 containing 10% fetal calf serum (FCS, Gibco), 2 mM L-glutamine, antibiotics (100 IU/mL penicillin and 100 µg/mL streptomycin), 1 mM sodium pyruvate, and 50 µM 2-mercaptoethanol. Cell culture media for S1P chemotaxis was same as above except charcoal-dextran filtered FCS was used.

### Flow cytometry, antibodies, and cell proliferation

Single cells were re-suspended in PBS, 2% FBS, and stained with fluorochrome-conjugated or biotinylated antibodies against B220 (RA3-6B2), IgD (11-26c-2a), IgM (R6-60.2), CD24 (M1/69), CD3 (145-2C11), CD4 (GK1.5), CD5 (53-7.3), CD8 (53-6.7), CD11c (HL3), CD11b (M1/70), CD138 (281-2), CD19 (1D3), CD38 (90), IgG1 (X56), CD93 (AA4.1), BP-1 (6C3), GL-7 (GL-7, Ly-77), CD95 (Jo2), CD21 (4E3), CD23 (B3B4), CD43 (S7), CD184 (CXCR4, 2B11), CXCR5 (2G8), CCR7 (4B12), CD11a (M17/4), CD29 (HMb1-1), CD49d (9C10, MFR4.B), CD54 (YN1/1.74), CD62L (MEL-16), α4β7 (DATK32), CD279 (PD-1, RMP1-30), CD45.1 (A20), or CD45.2 (104) (all from eBioscience, Biolegend, or BD Pharmingen). Biotin-labeled antibodies were visualized with fluorochrome-conjugated streptavidin (eBioscience). LIVE/DEAD® Fixable Aqua Dead Cell Stain Kit (Molecular Probes) was used in all experiments to exclude dead cells. Data acquisition was done on FACSCanto II (BD) flow cytometer and analyzed with FlowJo software (Treestar). The cell proliferation studies were performed using the eFluor® 450 (eBioscience) in a standard dye dilution assay. Purified B cells were stimulated for 96 hours with various combinations of the following reagents: 1 µg/ml CD40 (HM40-3), 1 µg/ml LPS (Sigma-Aldrich), recombinant mouse IL-4 (10 ng/ml, R&D Systems), or 10 µg/ml AffiniPure F(ab')_2_ fragment goat anti-mouse IgM (Jackson ImmunoResearch Laboratories). Data acquisition was done on FACSCanto II flow cytometer. The percent of cell divisions were calculated using FlowJo software, which is defined as the proliferation indexes divided by the division indexes, and multiplying the results by 100, assuming no cell death.

### Chemotaxis assays

Chemotaxis assays were performed using a transwell chamber (Costar) as previously described [22]. Splenic B cells were immunostained for B cell subsets with fluorochrome-conjugated antibodies against B220, CD21, CD23, CD24, CD93, IgM and IgD, washed twice, re-suspended in complete RPMI 1640 medium and added in a volume of 100 µl to the upper wells of a 24-well transwell plate with a 5 µm insert. Lower wells contained various doses of chemokines in 600 µl of complete RPMI 1640 medium. The numbers of cells that migrated to the lower well after 2 h incubation were counted using a MACSQuant flow cytometer (Miltenyi Biotec). The percent migration was calculated by the numbers of cells of a given subset that migrated into the bottom chamber divided by the total number of cells of that subset in the starting cell suspension, and multiplying the results by 100. D-erythro-sphingosine 1-phosphate was purchased from Avanti Polar Lipids. CXCL13, CCL19 and CXCL12 were purchased (R&D Systems). Fatty acid free bovine serum albumin (FAF-BSA) was purchased (Sigma-Aldrich).

### Intracellular calcium measurements

Cells were seeded at 10^5^ cells per 100 µl loading medium (RPMI 1640, 10% FBS) into poly-d-lysine coated 96-well black wall, clear-bottom microtiter plates (Nalgene Nunc). An equal volume of assay loading buffer (FLIPR Calcium 4 assay kit, Molecular Devices) in Hanks’ balanced salt solution supplemented with 20 mM HEPES and 2 mM probenecid was added. Cells were incubated for 1 h at 37 °C before adding chemokine or unconjugated AffiniPure F(ab')_2_ fragment goat anti-mouse IgM (Jackson ImmunoResearch Laboratories) and then the calcium flux peak was measured using a FlexStation 3 (Molecular Devices). The data was analyzed with SOFT max Pro 5.2 (Molecular Devices). Data is shown as fluorescent counts and the y-axis labeled as Lm1.

### Immunohistochemistry and immunocytochemistry

Freshly isolated spleens were snap frozen in Tissue-Tek OCT compound (Sakura Finetek). Frozen OCT splenic sections (8 µm) were acetone fixed for 2 min, and dried at room temperature. Slides were rehydrated in Tris-buffered saline (TBS) and stained in a humidified chamber in TBS/0.1% BSA/1% mouse serum overnight at 4°C or 1h. For immunohistochemistry primary antibodies included hamster anti-mouse CD3ε (145-2C11, purified), rat anti-mouse IgD (11-26c.2a, purified), rat anti-mouse CD35 (8C12, biotinylated), rat anti-mouse CD45R/B220 (RA3-6B2, purified), CD1d (1B1, rat anti-mouse biotin), and T and B cell activation antigen (GL7, rat anti-mouse FITC) all from BD Pharmingen. Donkey anti-mouse IgG F(ab')_2_ alkaline phosphatase and goat anti-mouse IgM F(ab')_2_ alkaline phosphatase were purchased (Jackson ImmunoResearch Laboratories). Anti-Ki67 (purified; rabbit polyclonal) and MadCAM (MECA 367, FITC) were from Abcam. Biotinylated-rat anti-mouse IgA was from Southern Biotech. Biotinylated antibodies were detected with streptavidin-alkaline phosphatase (Jackson ImmunoResearch Laboratories), FITC labeled antibodies were detected with anti-FITC-alkaline phosphatase or peroxidase (Roche), and purified mAbs with alkaline phosphatase or Horseradish peroxidase conjugated goat anti-Armenian hamster IgG (H+L), donkey anti-rabbit IgG (H+L), donkey anti-rat IgG (H+L), or goat anti-rabbit IgG (H+L), (Jackson ImmunoResearch Laboratories). Horseradish peroxidase was reacted with DAB (Peroxidase Substrate Kit; Vector), and alkaline phosphatase with Fast Blue/Naphthol AS-MX phosphate (Sigma-Aldrich). Peroxidase Block (Dako) or Levamisole (Vector Labs) were used to block endogenous alkaline phosphatase and peroxidase activities, respectively. Slides were mounted using Crystal Mount (Electron Microscopy Sciences). For immunofluorescence, frozen acetone fixed slides were stained with donkey anti-mouse IgG F(ab')_2_ Rhodamine-Red-X or a mixture of goat anti-mouse IgM F(ab')_2_ Dylight Alexa 488 (Jackson ImmunoResearch) and rat anti-mouse IgD (11-26c.2a, purified, BD Pharmingen), 4°C overnight. The IgD antibody was detected with donkey anti-rat IgG F(ab')_2_ Rhodamine-Red-X. Slides were mounted with Vectashield mounting media (Vector Labs). Images were acquired either with an Olympus BX-50 microscope equipped with a 4X or 10X objective and a Jenoptik-digital microscope camera. Fluorescent images were collected on inverted Leica TCS-SP5 confocal microscope equipped with an argon (488 nm) and Red HeNe (561 nm) (Leica Microsystems). A 4X, 10X, or 20X objective was used for imaging.

### Homing assays

Purified splenic B cells from *Gnai2* ^fl/fl^ mice, *Gnai2* ^*fl/fl*^ *mb-1Cre*, *Gnai3* ^*-/-*^ and *DKO* mice were labeled with 0.5 µM CMFDA, 1 µM eFluor® 670, 1 µM eFluor® 450, or 2.5 µM CMTMR for 15 min at 37°C and equal numbers of viable cells (8-10 million) were injected intravenously into recipient mice. After 2 h, spleen, iLNs, and pLNs were removed and gently dissociated into single cell suspensions. Peripheral blood was collected by retro-orbital eye bleeding. After removing red blood cells with Tris-NH_4_Cl, the cells were re-suspended in PBS containing 1% BSA at 4°C. LIVE/DEAD® Fixable Aqua Dead Cell Stain Kit (Molecular Probes) was used to exclude dead cells. Data acquisition was done on FACSCanto II flow cytometer and analyzed with FlowJo software (Tree Star).

### Intravital and spleen section two-photon laser scanning microscopy (TP-LSM)

Inguinal LNs were prepared for intravital microscopy as described [9,30]. Cell populations were labeled for 15 minutes at 37°C with 1 µM green cell tracker CMFDA, 2.5~5 µM red cell tracker CMTMR (Molecular probes), or blue cell tracker eFluor® 450 (eBioscience). 10-30 million labeled cells of each population in 200 ml of PBS were adoptively transferred by tail vein injection into 6~10-week-old recipient mice. After anesthetizing the mice by intraperitoneal injection of Avertin (300 mg/kg, tribromoethanol, Sigma), the skin and fatty tissue over inguinal LN were removed. The mouse was placed in a pre-warmed coverglass chamber slide (Nalgene, Nunc). The chamber slide was then placed into the temperature control chamber on the Leica SP5 microscope. The temperature of air was monitored and maintained at 37.0± 0.5°C. Inguinal LN was intravitally imaged from the capsule over a range of depths (10-220 µm). A freshly made spleen slice was fitted into a coverglass chamber slide and imaged from the surface to a 100 µm depth. All of Two-photon imaging was performed with a Leica SP5 inverted 5 channel confocal microscope (Leica Microsystems) equipped with 20x multi-immersion objective, 0.7 NA (immersion medium used 80% glycerol) or 25x water dipping objective, 0.95 NA (immersion medium used distilled water). Two-photon excitation was provided by a Mai Tai Ti: Sapphire laser (Spectra Physics) with a 10 W pump, tuned to 810 or 910 nm. Emitted fluorescence was collected using a 4 channel non-descanned detector. Wavelength separation was through a dichroic mirror at 560 nm and then separated again through a dichroic mirror at 495 nm followed by 460/50 nm emission filter for second harmonics or eFluor® 450 (eBiosciences); 525/50 emission filter for CMFDA (Molecular probes); a dichroic mirror at 650 nm followed by 610/60 nm emission filter for CMTMR; and the evans blue signal was collected by 680/50 nm emission filter. Sequences of image stacks were transformed into volume-rendered four-dimensional videos using Imaris software v.7.5.0 or v.7.5.2 (Bitplane).

### Immunizations and ELISAs

WT and mutant mice were immunized with either sheep RBCs, NP_35_-KLH, or NP_40_-Ficoll. For the sheep RBC immunizations 200 µl of 10% solution of sheep RBCs (Lonza Walkerville, Inc.) was given by intraperitoneal injection. NP_35_-KLH (Biosearch Technology) was mixed with Imject® Alum (Thermo Scientific) and introduced into mice (100 µg) via intraperitoneal injection. Mice were boosted with same dose of antigen at the indicated days along with Alum. Mice were also immunized with 25 µg NP_40_-Ficoll (Biosearch Technologies) via intraperitoneal injection and boosted with same dose of antigen at the indicated days. Serum NP specific Ig levels in these mice were analyzed by ELISA. Briefly, 96 well ELISA plates (Nunc) were coated with NP_5_-BSA or NP_30_-BSA (Biosearch Technology) overnight at 4^o^C, washed and blocked with 1% BSA fraction V (Sigma-Aldrich), serum titers were then added to the plates and incubated 4 h at 4^o^C. After washing alkaline phosphatase-labeled goat anti-mouse Ig isotype specific antibodies were added for 2 h at RT (SouthernBiotech). After washing, PNPP one component substrate (SouthernBiotech) was used to detect the amount of secondary antibody bound.

### Statistics

*In vivo* results represent samples from 3–9 mice per experimental group. Results represent mean values of at least triplicate samples. Standard errors of the mean (SEM) and *p* values were calculated with *t* test or 2 way ANOVA using Microsoft Excel software or GraphPad Prism (GraphPad software).

## Results

### Loss of B cells in the Gnai2 ^fl/fl^mb1-cre mice is exacerbated in the DKO mice

**Figure 1:**
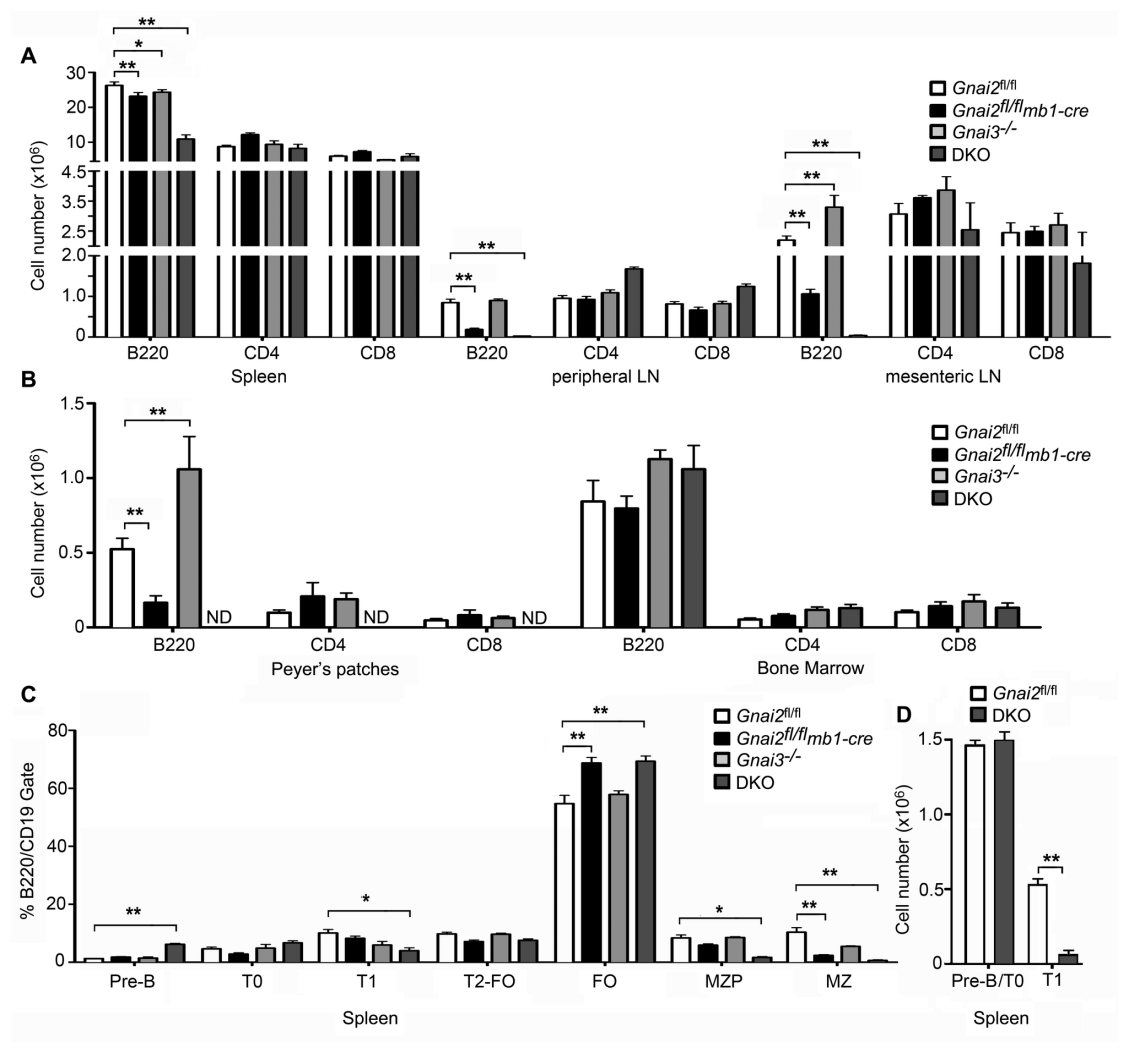
Reduced numbers of B cells in the *Gnai2* ^fl/fl^ *mb1-cre* mice and DKO mice. A. Flow cytometric analysis of lymphocytes in the spleen and LNs of control and mutant mice. Pooled results from analyzing cells prepared from spleens and LNs from 9 *Gnai2* ^fl/fl^, 8 *Gnai2* ^fl/fl^ *mb1-cre*, 3 Gnai3^-/-^, and 3 DKO mice. B. Flow cytometric analysis of lymphocytes in Peyer’s patches and bone marrow of control and mutant mice. Pooled results from analyzing cells from Peyer’s patches and bone marrow from the same mice used in part A. ND- not detected. C. Flow cytometric analysis of B cell populations in the spleen of control and mutant mice. Pooled results from analyzing cells from the spleens prepared from the same mice used in part A. D. Flow cytometric analysis of immature B cell populations in the spleen. The number of pre-B and T0 B cells pooled and compared to T1 B cells. Data is mean +/- SEM. Statistical significance determined by 2 way ANOVA. ** p<0.001, * p<0.05.

The *Gnai2* ^fl/fl^ *mb1-cre* and the DKO mice have a single normal *mb1* (CD79a) allele as the other allele has been disrupted by insertion of the coding region for the *cre* recombinase. A single *mb1* allele suffices for normal B cell function [29]. Initially, we compared the lymphocyte populations in various lymphoid organs from the *Gnai2* ^fl/fl^ mice (control) to the mice missing Gα_i2_ and/or Gα_i3_ in their B lymphocytes. The absence of Gα_i2_ reduced the number of B lymphocytes in the peripheral lymph nodes (LN), mesenteric LNs, and Peyer’s patches without disturbing the number of splenic B cells. The loss of Gα_i3_ had a small impact on B cell compartments. We noted a slight decrease in B220^+^ cells in the spleen and slightly larger mesenteric lymph nodes and Peyer’s patch in these mice, which was reflected increased numbers of B220+ cells at those sites (Figure 1A & B). The loss of Gα_i3_ in B cells that lack Gα_i2_ caused a profound loss of B cells from lymph nodes and Peyer’s patches; and a 50-60% reduction of splenic B cells. Despite the loss of splenic B cells, the DKO mice had normal numbers of splenic CD4 and CD8 T cells. Surprisingly, the *Gnai2* ^fl/fl^ *mb1-cre* and DKO mice also had unperturbed numbers of bone marrow B cells (Figure 1A & B). To address the reduction in spleen B220^+^ cells in the DKO mice and to assess whether marginal zone B cell development was impaired as had been noted in the Gnai2^-/-^ mice [7], we examined the relative distribution of the B cell populations in the spleen in the different mice. Revealing a B cell intrinsic role for Gα_i2_ in marginal zone B cell development, the *Gnai2* ^fl/fl^ *mb1-cre* mice had a significant decrease in marginal zone B cells (Figure 1C). Otherwise the splenic populations were not significantly altered. The splenic B cell populations in the Gnai3^-/-^ mice resembled those of the control mice while those of the DKO mice had a nearly complete loss of marginal zone B cells, but also a disturbance in splenic B cell maturation. When analyzed as percentages of the B220^+^/CD19^+^ gate the B cell populations in the DKO spleens had an increase in pre-B cells and reduction in T1 B cells (Figure 1C). Correcting for the reduced B cell recovery from DKO mice spleens revealed similar numbers of immature (pre-B plus T0) cells as were present in control mice, but a marked reduction in the number of T1 B cells (Figure 1D). Nevertheless, mature follicle B cells developed in the DKO spleens. These results indicate that the B cell specific loss of Gα_i2_ reduced B cell populations in lymph nodes and Peyer’s patches, and the marginal zone of the spleen. The additional loss of Gα_i3_ nearly eliminates Peyer’s patch, LN, and splenic marginal zone B cells. While follicular B cell differentiation occurs the absolute number of B cells in the spleen is less than half of normal. The severe reduction in T1 B cells suggests a partial block in immature B cell differentiation.

### Bone marrow development and egress is perturbed in the Gnai2 ^fl/fl^mb1-cre and DKO mice

**Figure 2:**
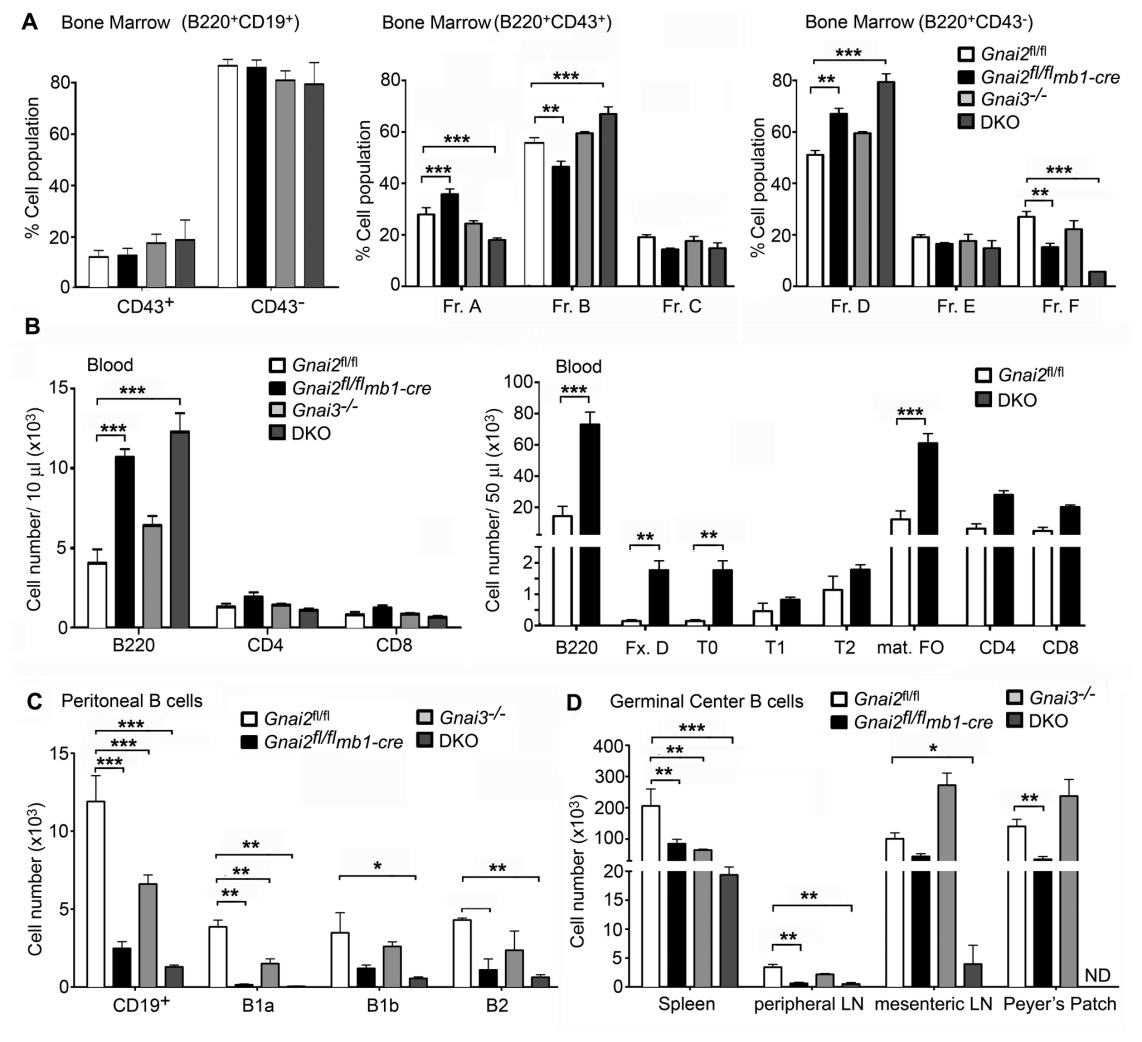
Disturbed B cell development and trafficking in *Gnai2* ^fl/fl^ *mb1-cre* and DKO mice. A. Flow cytometric analysis of bone marrow B cells fractions in the control and mutant mice. Pooled results from analyzing bone marrow from at least 6 mice of each genotype. B. Flow cytometric analysis of lymphocytes and B cell population in the blood of control and mutant mice. Pooled results from analyzing blood from 6 *Gnai2* ^fl/fl^, 4 *Gnai2* ^fl/fl^ *mb1-cre*, 4 Gnai3^-/-^, and 6 DKO mice. C. Flow cytometric analysis of B cell populations in the peritoneum of control and mutant mice. Same mice as used in part B. D. Flow cytometric analysis of spontaneous GCs in control and mutant mice. Pooled results from analyzing cells prepared from spleens and LNs from 9 *Gnai2* ^fl/fl^, 8 *Gnai2* ^fl/fl^ *mb1-cre*, 3 Gnai3^-/-^, and 3 DKO mice. Data is mean +/- SEM. Statistical significance determined by 2 way ANOVA. *** p<0.001, ** p<0.01, * p<0.05.

The relatively normal number of B220^+^ B cells in the bone marrow and immature B cells in the spleen suggested that bone marrow development and egress of B cells was not severely disrupted in the DKO mice. To assess bone marrow B cell development we isolated bone marrow from the different mice and analyzed B cell precursors based on Hardy’s fractionation method [31]. Overall B cell development was intact although we found some disturbances in the differentiation of B cell progenitors. The different mice had similar numbers of B220 ^+^ CD43^+^ and B220 ^+^ CD43^-^ (Figure 2A, left panel), but further analysis of the B220 ^+^ CD43^+^ cells revealed that the *Gnai2* ^fl/fl^ *mb1-cre* mice had an increase in Fraction (Fr.) A and a decrease in Fr. B cells compared to the control and Gnai3^-/-^ mice while the DKO mice had a reduction in Fr. A and an increase in Fr. B cells. Yet the numbers of Fr. C cells in all the mice were similar (Figure 2A, middle panel). The explanation for the reciprocal changes in Fr. A and Fr. B cells is unclear and counter to most of the other phenotypes, where the DKO mice have an exaggerated phenotype of that found in the *Gnai2* ^fl/fl^ *mb1-cre* mice. Further analysis of the B220 ^+^ CD43^-^ cells in the different mice revealed an increase in Fr. D (pre-B) cells and a reduction in Fr. F (mature recirculating) cells in the bone marrow of the *Gnai2* ^fl/fl^ *mb1-cre* mice, which was more apparent in the DKO mice (Figure 2A, right panel). The accumulation of Fr. D cells in the DKO mice implies either an abnormal expansion of late pro-B cells or a problem at the transition from pre-B cells to immature B cells. Yet the number of Fr. E (immature B) cells in the bone marrow was similar to the control and Gnai3^-/-^ mice. To assess whether immature B cells were being released into the circulation we quantitated the numbers of immature and mature B cells in the blood. The overall number of B cells in the blood of the *Gnai2* ^fl/fl^ *mb1-cre* and DKO mice were significantly elevated (Figure 2B, left panel). Consistent with an early release from the bone marrow the DKO mice had a marked increase in Fr. D (pre-B cells) and T0 B cells in their blood (Figure 2B, right panel). These results show that despite a likely impaired CXCR4 bone marrow retention signal [11] and the S1P egress signal [13], B cells develop in the bone marrow and can leave the bone marrow to access the blood.

### Reduced trafficking of B cells to the peritoneum and decreased germinal center B cells in the Gnai2 ^fl/fl^mb1-cre and DKO mice

The CXCL13/CXCR5 axis play a dominant role in recruiting cells to the peritoneum and other body cavities [32]. To test the relative roles of *Gnai2* and *Gnai3* in the trafficking of B cells to the peritoneum, we assessed the numbers and phenotypes of the B cells in the peritoneum of our different mice. In the *Gnai2* ^fl/fl^ *mb1-cre* mice we found an 80% reduction in B cells while in the DKO mice had a 90% reduction (Figure 2C). Surprisingly, the Gnai3^-/-^ mice also had a significant decrease in peritoneal B cells suggesting that optimal entrance of B cells into the peritoneum requires both Gα_i2_ and Gα_i3_. The B1a subset of cells was most severely affected in each of the mutant mice and were nearly absent in the DKO mice (Figure 2C). Despite the severe loss of B1 cells in the peritoneum of the DKO mice, we found similar numbers of B1a and B1b in spleens from DKO and wild type mice (IYH, unpublished data). Next, we characterized spontaneous germinal center (GC) B cells at various sites. We detected fewer GC B cells in the spleens of the mutant mice and as expected the DKO mice were most affected. There was also a reduction of GC B cells in the Peyer’s patches of the *Gnai2* ^fl/fl^ *mb1-cre* mice. Consistent with the severe reduction of B cells in lymph nodes and the loss of Peyer’s patches GC B cells were severely reduced in the DKO mice at those sites (Figure 2D). These data indicate that the lack of Gα_i3_ exacerbates the already severe reduction in B1 cells observed in the mice lacking B cell specific expression of Gα_i2_ and that constitutive GC formation is reduced in the absence of Gα_i2_ and Gα_i3_ signaling.

### Splenic B cell B cell antigen receptor (BCR) signaling is impaired in the Gnai2 ^fl/fl^mb1-cre and DKO mice

**Figure 3:**
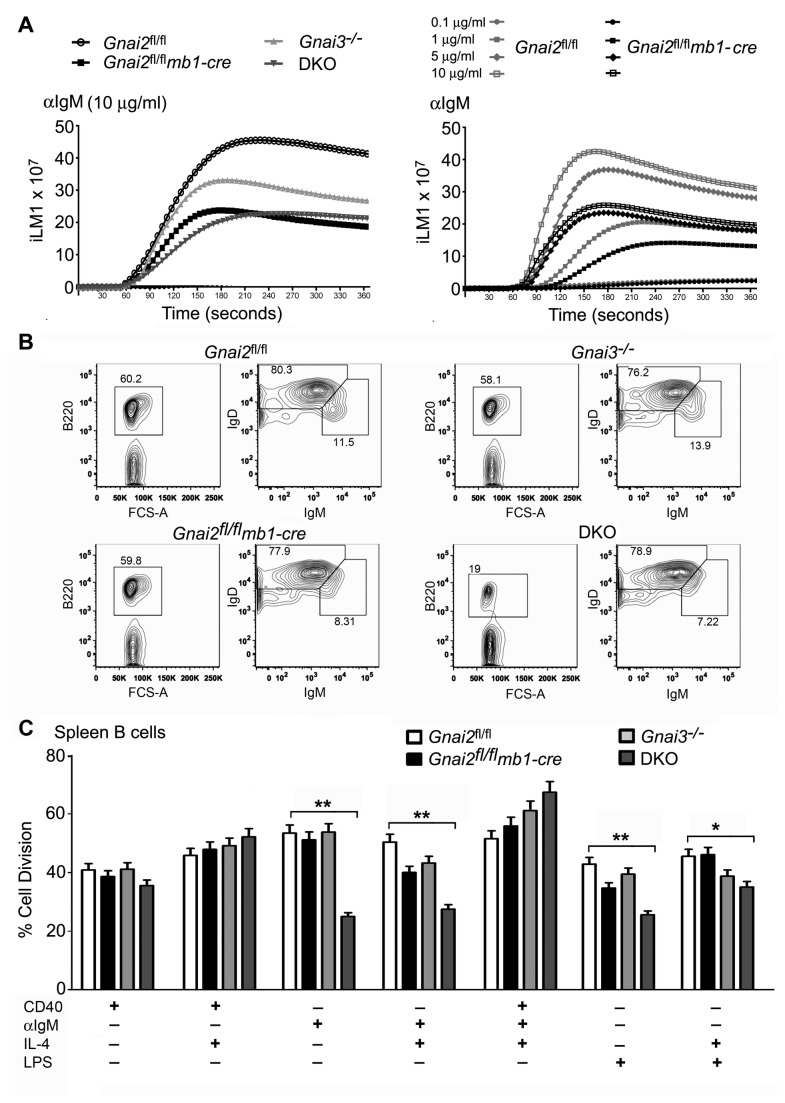
Impaired BCR signaling and reduced proliferation of DKO B cells to both anti-IgM and LPS. A. Intracellular calcium response to anti-IgM using control and mutant B cells. *Gnai2* ^fl/fl^, *Gnai2* ^fl/fl^ *mb1-cre*, Gnai3^-/-^, and DKO B cells were stimulated with anti-IgM and the induced changes in intracellular calcium were monitored over 3 minutes, left panel. The data shown as fluorescent counts and the y-axis labeled as iLm1. Each experimental value is the mean of three determinations. Similar experiment comparing the intracellular calcium responses of B cells prepared from *Gnai2* ^fl/fl^ or *Gnai2* ^fl/fl^ *mb1-cre* mice to increasing concentrations of anti-IgM, right panel. Experiments repeated three times with similar results. B. Flow cytometry results from the analysis of B220, IgD, and IgM expression on spleen B cells from *Gnai2* ^fl/fl^, *Gnai2* ^fl/fl^ *mb1-cre*, Gnai3^-/-^, and DKO B cells. For each genotype B220 expression is shown on cells in the lymphocyte gate and a second plot of IgM versus IgD expression after gating on B220^+^ cells. Data is representative of multiple experiments. C. Dye dilution assay to assess the initial proliferative potential of wild type and mutant B cells. B cells from control and mutant strains were stained with Pacific blue and then cultured with the indicated stimulants for 96 h. The percentage of cells that divided in each cultured was determined by the analysis of flow cytometry data. The results are from the analysis of B cells from two mice of each genotype. Statistics are from analysis of the data by 2 way ANOVA. ** p<0.001, * p<0.05.

Our results indicate that normal marginal zone B cell development requires Gα_i_ signaling in B lymphocytes. Compared to the *Gnai2* ^fl/fl^ mice the splenic B cell compartment in the DKO mice had a 5- and 15-fold reduction in the % of B cells that had a marginal zone precursor or a marginal zone B cell phenotype, respectively. The initial studies of the Gnai2^-/-^ mice reported a 3-fold reduction in the % of marginal zone B cells compared to controls [7]. Arguing for an important role for Gα_i3_ in marginal zone B cell development the DKO mice had more than a 30 fold reduction in the absolute number of marginal zone B cells. Signaling through the B cell antigen receptor and Notch2 has been linked to the follicular/marginal zone B cell fate decision [33]. Mutations in components of the BCR signaling pathway that reduce signal transduction promote a marginal zone rather than a follicular B cell fate [34]. B cells isolated from the original Gnai2^-/-^ mice had an enhanced intracellular calcium response to anti-IgM stimulation suggesting that functionally Gα_i2_ suppressed BCR signaling [7]. However, we consistently found that the B cells from the *Gnai2* ^fl/fl^ *mb1-cre* and DKO mice had diminished intracellular calcium responses to anti-IgM stimulation (Figure 3A). The reason for this discrepancy is unclear although the genetic backgrounds of the mice are different. It was also not secondary to the loss of an *mb-1* allele in the Gnai2 ^fl/fl^ *mb1-*cre mice as B cells from *mb1-cre* and *Gnai2* ^fl/fl^ mice responded similarly to BCR crosslinking (IYH, unpublished observation). These data indicate that the loss of marginal zone B cells in mice lacking Gα_i2_ expression in their B cells is unlikely related to altered BCR signaling

Reduced B cell IgM expression cannot account for the decreased intracellular calcium response as IgM and IgD levels were not appreciably different on the splenic B cells from the different mice (Figure 3B). The IgM versus IgD flow cytometry plots reveals the loss of marginal zone B cells in the *Gnai2* ^fl/fl^ *mb1-cre* and the DKO mice as the IgM ^high^IgD^low^ cells are reduced or missing. In addition, we consistently found that the DKO B cells had a lower level of B220 expression (Figure 3B). B220 levels normally increase during B cell differentiation however this was much less evident on the DKO B cells despite their acquisition of IgD. Indicating that the reduced intracellular calcium response to IgM crosslinking affected B cell function, the DKO B cells divided less well following IgM crosslinking (Figure 3C). This was not an intrinsic defect in the capacity of the DKO cells to divide as they responded similar to wild type B cells when stimulated via CD40 crosslinking. The DKO B cells also divided less well to LPS stimulation presumably due to the loss of marginal zone B cells (Figure 3C). These results suggest enhanced BCR signaling does not explain the loss of marginal zone B cells in the *Gnai2* ^fl/fl^ *mb1-cre* and DKO mice, but rather it likely results from a failure of B cells to receive a GPCR signal needed for marginal zone B cell development perhaps by modulating the Notch2 signaling pathway. These results also suggest that the lack of signaling through Gα_i_ directly or indirectly impacts BCR signaling.

### DKO B cells fail to migrate to chemoattractants while B cell subsets lacking Gα_i2_ exhibit uniformly poor responses to standard B cell chemokines

**Figure 4:**
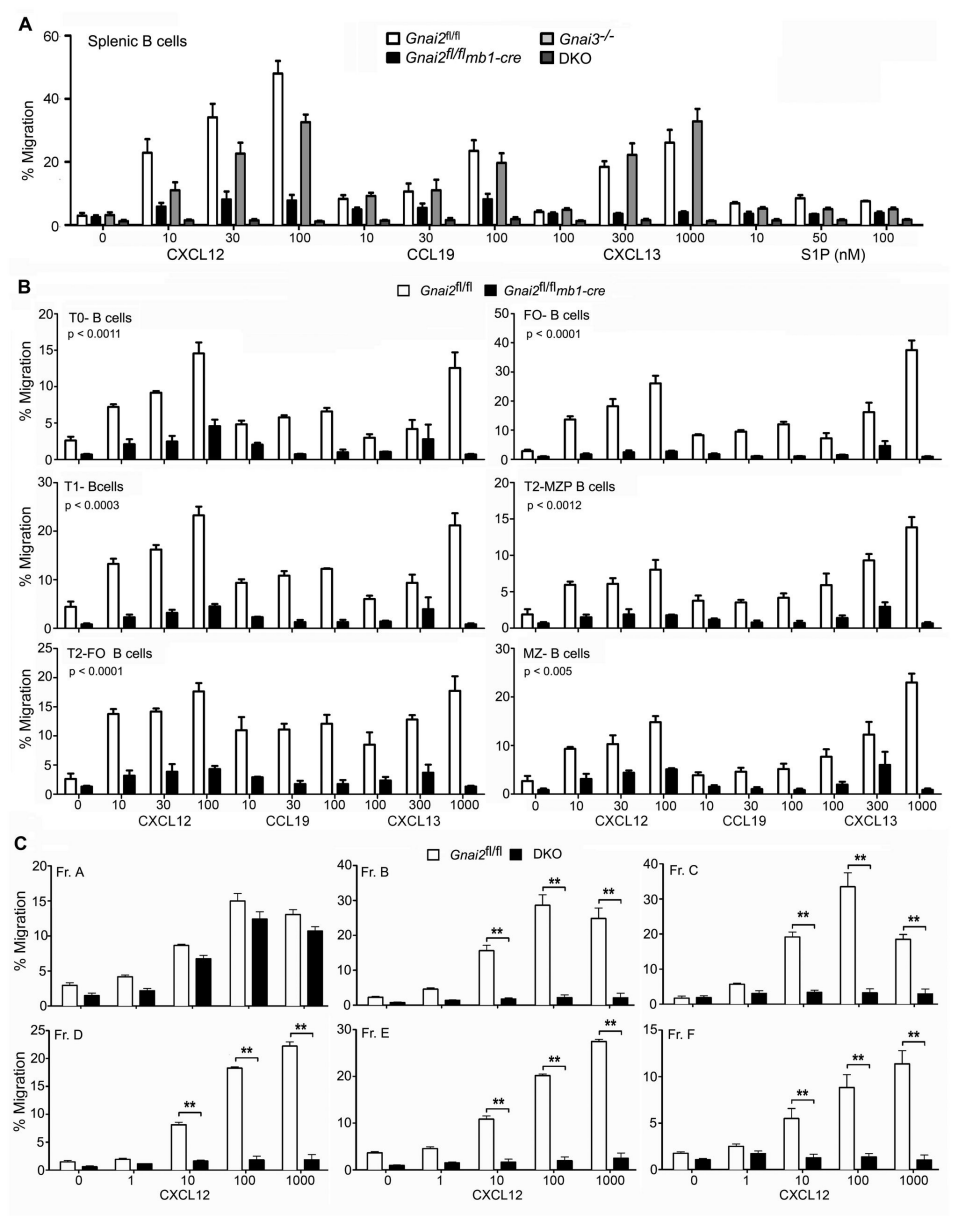
*Gnai2* ^fl/fl^ *mb1-cre* B cells migrate poorly to chemoattractants and DKO B cells are refractory. A. *In vitro* chemotaxis assay using splenic B cells from control and mutant mice. Splenocytes were from 3 mice of each genotype and the assay was performed in duplicate. At each concentration the specific migration of *Gnai2* ^fl/fl^ *mb1-cre* and DKO B cells were statistically different from that of wild type mice. The indicated chemokine concentrations are ng/ml. Data shown as mean +/- SEM. B. *In vitro* chemotaxis assay using splenic B cells immunostained to allow distinction of various B cells subsets from control and *Gnai2* ^fl/fl^ *mb1-cre* mice. Splenocytes were prepared from 3 mice of each genotype and the assay performed in duplicate. The specific chemokine and concentration (ng/ml) is indicated. At each concentration the specific migration of *Gnai2* ^fl/fl^ *mb1-cre* B cells was statistically different from that of control mice. Data shown as mean +/- SEM. The p values are from a paired *t* test comparing results between B cell subsets from control and *Gnai2* ^fl/fl^ *mb1-cre* mice. C. *In vitro* chemotaxis assay was performed using partially purified bone marrow B cells immunostained to allow distinction of different developmental B cell fractions from control and DKO mice. The bone marrow cells were prepared from 3 mice of each genotype. Following depletion of Gr1, Ter119, CD11b, Sca1 and c-kit positive cells, the remaining cells were immunostained for B220, CD43, CD24, BP-1, IgM, and IgD and subjected to a chemotaxis assay. Assay performed in triplicate. The indicated concentrations of CXCL12 were added to the bottom wells. Data shown as mean +/- SEM and the statistics performed by 2 way ANOVA. ** p < 0.001.

B cells isolated from Gnai2^-/-^ mice have reduced responses to chemoattractants [8]. To test whether their residual responsiveness depended upon Gα_i3_, we compared the chemotatic responses of B cells isolated from the *Gnai2* ^fl/f^, *Gnai2* ^fl/fl^ *mb1-cre*, Gnai3^-/-^, and DKO mice. Using standard chemotaxis assays we checked their responses to varying concentrations of CXCL12, CCL19, CXCL13, and S1P. The *Gnai2* ^fl/fl^ *mb1-cre* B cell chemotaxis had a significant reduction in their responses to all the chemoattractants that varied between 70 and 90% (Figure 4A). The Gnai3^-/-^ B cells had a slightly reduced response to CXCL12, similar to CCL19, a slightly heightened response to CXCL13, but a reduced one to S1P indicating that Gα_i2_ largely compensated for the loss of Gα_i3_. Analysis of the DKO B cells showed that Gα_i3_ clearly contributes to B cell chemoattractant responses as the DKO B cells were essentially refractory to all the chemoattractants (Figure 4A). We also tested whether different splenic B cell subsets varied in their dependence upon Gα_i2_ for directed migration. This showed that all the splenic B cell subsets depended upon Gα_i2_ for their migration. Of the different subsets, follicular B cells were the most dependent (Figure 4B). To determine when during B cell development the *mb1-cre* mediated *Gnai2* deletion became functionally relevant, we performed chemotaxis assays using bone marrow cells prepared from control and DKO mice. This analysis revealed that the loss of *Gnai2* expression correlated with the known expression pattern of *mb1* as bone marrow Fr. A cells responded to CXCL12, while Fr. B and more mature cells did not (Figure 4C). Overall these data indicate that B cells chemoattractant receptors largely use Gα_i2_ to trigger chemotaxis. In its absence B cells can to a limited degree use Gα_i3_. The absence of both proteins leads to a profound loss of chemoattractant signaling.

### Proximal chemoattractant receptor signaling and in vivo trafficking of adoptively transferred B cells shows the same dependence on Gα_i2_ and Gα_i3_

**Figure 5:**
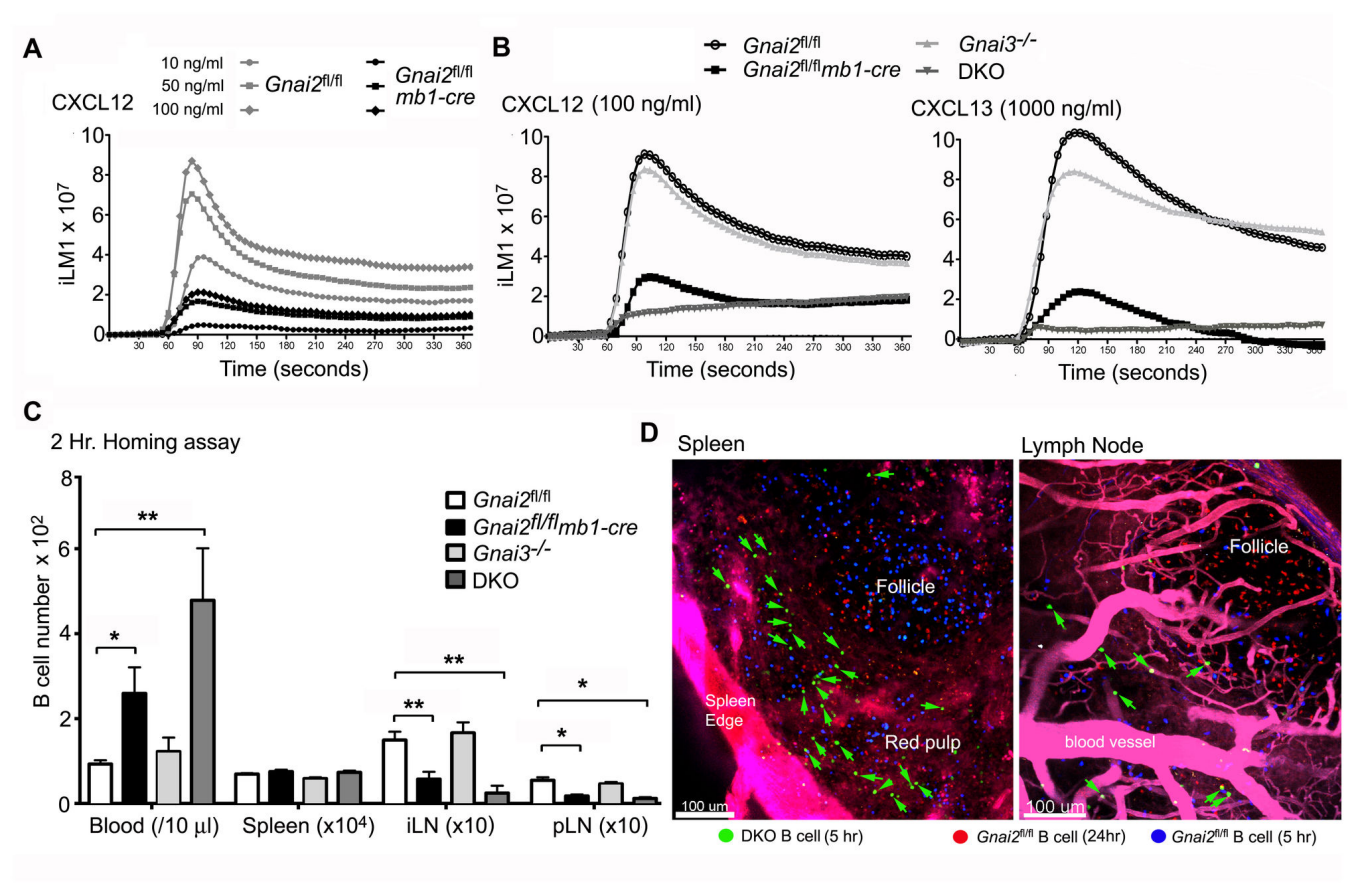
Further defects in chemoattractant signaling in the *Gnai2* ^fl/fl^ *mb1-cre* and DKO mice. A. Changes in intracellular calcium were monitored over 3 minutes in B cells from control and Gnai2 ^fl/fl^ *mb1-cre* mice exposed to increasing concentrations of CXCL12. The data shown as fluorescent counts and the y-axis labeled as iLm1. Each experimental value is the mean of three determinations. Similar results using B cells prepared from 3 mice of each genotype. B. Changes in intracellular calcium were monitored over 3 minutes using B cells from control and mutant mice exposed to CXCL12 or CXCL13. The data shown as fluorescent counts and the y-axis labeled as iLm1. Data from B cells prepared from 2 mice of each genotype. C. *In vivo* homing assay using B cells from control and mutant mice. B cells from each genotype were fluorescently labeled and adoptively transferred to a wild type recipient. The cells in the blood, spleen, inguinal and axillary LNs (pLN), and the mesenteric LNs were enumerated by flow cytometry. Data shown as mean+/- SEM. Data analyzed by *t* test. D. Images from multiphoton microscopy of the spleen and inguinal LN following the adoptive transfer of control and DKO B cells. Fluorescently labeled *Gnai2* ^fl/fl^ B cells were adoptively transferred 5 h (blue) and 24 h (red) prior to imaging. The DKO B cells (green) were transferred 5 h prior to imaging. The LN was imaged intravitally while a fresh section was used for the spleen imaging. Evans blue was infused intravenously immediately before the imaging to outline blood vessels. ** p<0.001, * p<0.01.

Next, we checked proximal chemoattractant receptor signaling using splenic B cells prepared from the same mice. Chemoattractant receptor signaling triggers a rapid increase in intracellular calcium. Gα_i_ nucleotide exchange releases Gβγ subunits, which leads to the generation of inositol 1,4,5-trisphosphate [35]. This causes the release of calcium from intracellular stores, which can be measured by monitoring intracellular calcium levels using calcium sensitive fluorescent probes. B cells prepared from the *Gnai2* ^fl/fl^ and *Gnai2* ^fl/fl^ *mb1-cre* mice were exposed to different concentrations of CXCL12. At each concentration tested we found a 4-5 fold reduction in the peak intracellular calcium response (Figure 5A). When we substituted CXCL13 or CCL19 for CXCL12, we observed similar results (data not shown). Comparing B cells prepared from each of the strains to a single near optimal concentration of either CXCL12 or CXCL13 revealed that the Gnai3^-/-^ B cells behaved like control cells, the Gα_i2_ deficient B cells again had a 4-5 fold reduction in their peak response, and the DKO B cells failed to raise their intracellular calcium following exposure (Figure 5B). We also noted that the loss of Gα_i2_ or Gα_i2_ and Gα_i3_ modestly reduced the expression of CXCR4, CCR7, CXCR5, and several integrins (Table 1). This suggests that basal chemoattractant receptor signaling supports the expression of these receptors.

**Table 1:** Expression of chemokine receptor and integrins on B cells from different genotype mice.

Genotype^1^	CXCR4	CXCR5	CCR7	CD49d	CD11a	CD29	CD62L	α4β7	CD54
*Gnai2* ^fl/fl^	3405	3227	477	6128	13217	5065	5068	1850	8952
*Gnai2* ^fl/fl^ *mb1-cre*	2480	2051	419	5978	11978	5445	5500	1459	10149
Gnai3^-/-^	3215	3349	447	6629	11175	6351	5055	2172	8747
DKO	1828	2023	379	4879	8640	4198	4896	1414	10066

^1^Results are from the analysis of 3 mice or each genotype and shown as geometric means.

To determine the relative importance of Gα_i2_ and Gα_i3_ for *in vivo* chemoattractant receptor signaling, we compared the ability of B cells from the different mice to home to LNs by differentially fluorescently labeling B cells from the four different genotypes and adoptively transferred them simultaneously into *Gnai2* ^fl/fl^ mice. Two hours after transfer we collected the LNs, spleen, and blood and analyzed the recovered cells by flow cytometry. The *Gnai2* ^fl/fl^ *mb1-cre* B cells homed poorly to LNs accumulating in the blood, the DKO B cells exhibited even less capacity to enter LNs, while the Gnai3^-/-^ B cells homed normally (Figure 5C). To examine the localization of the transferred DKO cells in lymphoid organs we differentially labeled control and DKO cells, transferred them to control mice, and used multiphoton microscopy to assess their locations. In the spleen the DKO cells accumulated in the red pulp failing to enter the B cell follicle (Figure 5D, left panel). In LNs we detected very few DKO B cells and those that we found localized near blood vessels, not in the LN follicle. They also exhibited little or no motility and a non-polarized morphology (Figure 5D, right panel, data not shown). These results extend the *in vitro* chemotaxis data to confirm the importance of Gα_i2_ and Gα_i3_ for *in vivo* B cell trafficking.

### Gnai2 ^fl/fl^mb1-cre mice generate a poor, disorganized antigen-induced GC response while DKO mice lack antigen-induced GCs

**Figure 6:**
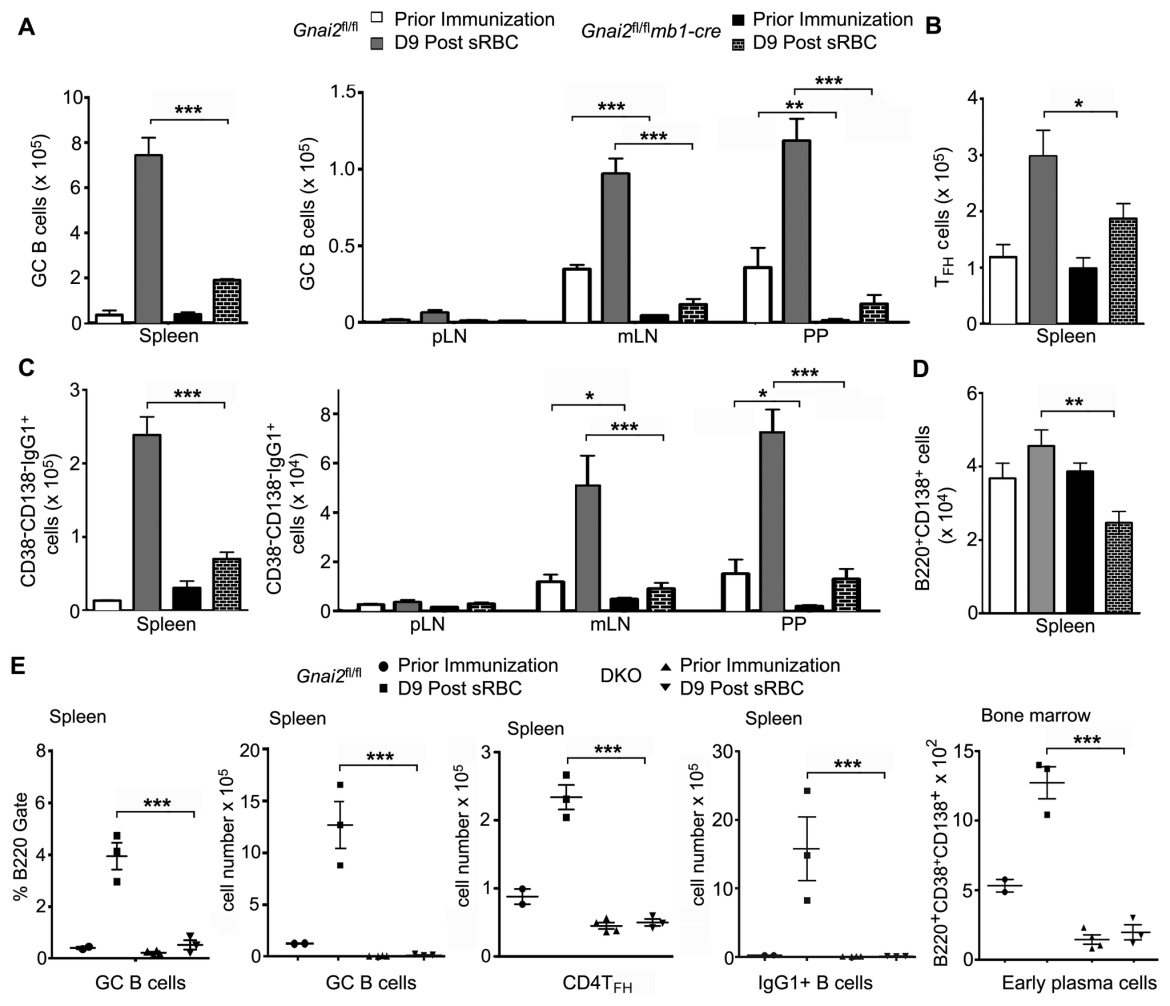
Defective antigen induced B cell responses in the *Gnai2* ^fl/fl^ *mb1-cre* and DKO mice. A. Flow cytometric analysis to enumerate GC B cells at various sites in control and *Gnai2* ^fl/fl^ *mb1-cre* mice following sRBC immunization. GC B cells were defined as B220 ^+^ CD38^-^GL7 ^+^ Fas^+^. B. Flow cytometric analysis of T_FH_ cells. T_FH_ cells were defined as CD4^+^CXCR5 ^+^ PD-1^+^. C. Flow cytometric analysis of antigen induced switched B cells at various sites. The numbers of B220 ^+^ CD38^-^IgG1^+^ cells were enumerated. D. Flow cytometric analysis of early splenic plasma cells. The numbers of B220 ^+^ CD138^+^ cells were determined. For the above experiments B cells were prepared from the indicated sites from 3 control and 3 *Gnai2* ^fl/fl^ *mb1-cre* mice and analyzed prior to immunization. Another set of mice were analyzed 9 days after intraperitoneal injection of sRBC. Statistics from an analysis of the data by *t* test. E. Flow cytometric analysis of antigen induced changes in splenic B cell population in control and DKO mice. Similar experiments as above with the exception that bone marrow plasma cells were assessed. Results are from mice reconstituted with *Gnai2* ^fl/fl^ or DKO bone marrow analyzed prior to and 9 days after sheep RBC immunization. Data is shown as mean +/- SEM and analyzed by *t* test. *** p<0.001, ** p<0.01, * p<0.05.

To address the impact on GC development of the loss of Gα_i2_ expression in B cells, we quantitated the GC B cells (B220 ^+^ CD38^-^GL7 ^+^ Fas^+^) induced in the spleen, peripheral LNs, mesenteric LNs, and Peyer’s patches of the *Gnai2* ^fl/fl^ *mb1-cre* mice 9 days following sheep RBC immunization. We also enumerated the number of IgG1^+^ B cells and CD138^+^ cells. We found that sheep RBC immunization induced GC B cell formation was reduced 4-fold in the spleen and more so in LNs and Peyer’s patches (Figure 6A). Following immunization, the T_FH_ cells (CD4^+^CXCR5 ^+^ PD-1^+^) expanded in both control and *Gnai2* ^fl/fl^ *mb1-cre* mice, but by a third less in the mutant mice (Figure 6B). The *Gnai2* ^fl/fl^ *mb1-cre* mice also had fewer early isotype switched B cells (B220 ^+^ CD38^-^CD138^-^IgG1^+^) and reduced number of early plasma cells (B220 ^+^ CD138^+^) in the spleen and at the other lymphoid sites (Figure 6C and 6D). To assess antigen induced GC formation in the DKO mice we used bone marrow reconstituted CD45.1 mice that had received bone marrow from either wild type (CD45.2) or DKO (CD45.2) mice. These mice lacked Gα_i2_ and Gα_i3_ in their B cells, Gα_i3_ in other bone marrow derived cells, while non-bone marrow derived cells had unperturbed Gα_i_ protein levels. When examined 8 weeks after reconstitution the B cell compartment had recovered normally in the mice that had received bone marrow from the *Gnai2* ^fl/fl^ mice while those that had received DKO bone marrow had a reduction in B220^+^ cells and a loss of marginal zone B cells as we had noted with the DKO mice. In addition, the DKO reconstituted mice essentially lacked LN B cells and had no visible Peyer’s patches. Immunization elicited few GC B cells, isotype switched B cells, or plasma cells (Figure 6E). Similar results were obtained with non-reconstituted DKO mice (data not shown). These data indicate that the lack of Gα_i2_ in B cells impairs antigen induced B cell responses while the loss of Gα_i2_ and Gα_i3_ leads to a profound quantitative and qualitative B cell immunodeficiency.

### The B cell compartments in the spleen, at mucosal sites, and in LNs are disrupted in Gnai2 ^fl/fl^mb1-cre mice and severely compromised in the DKO mice

**Figure 7:**
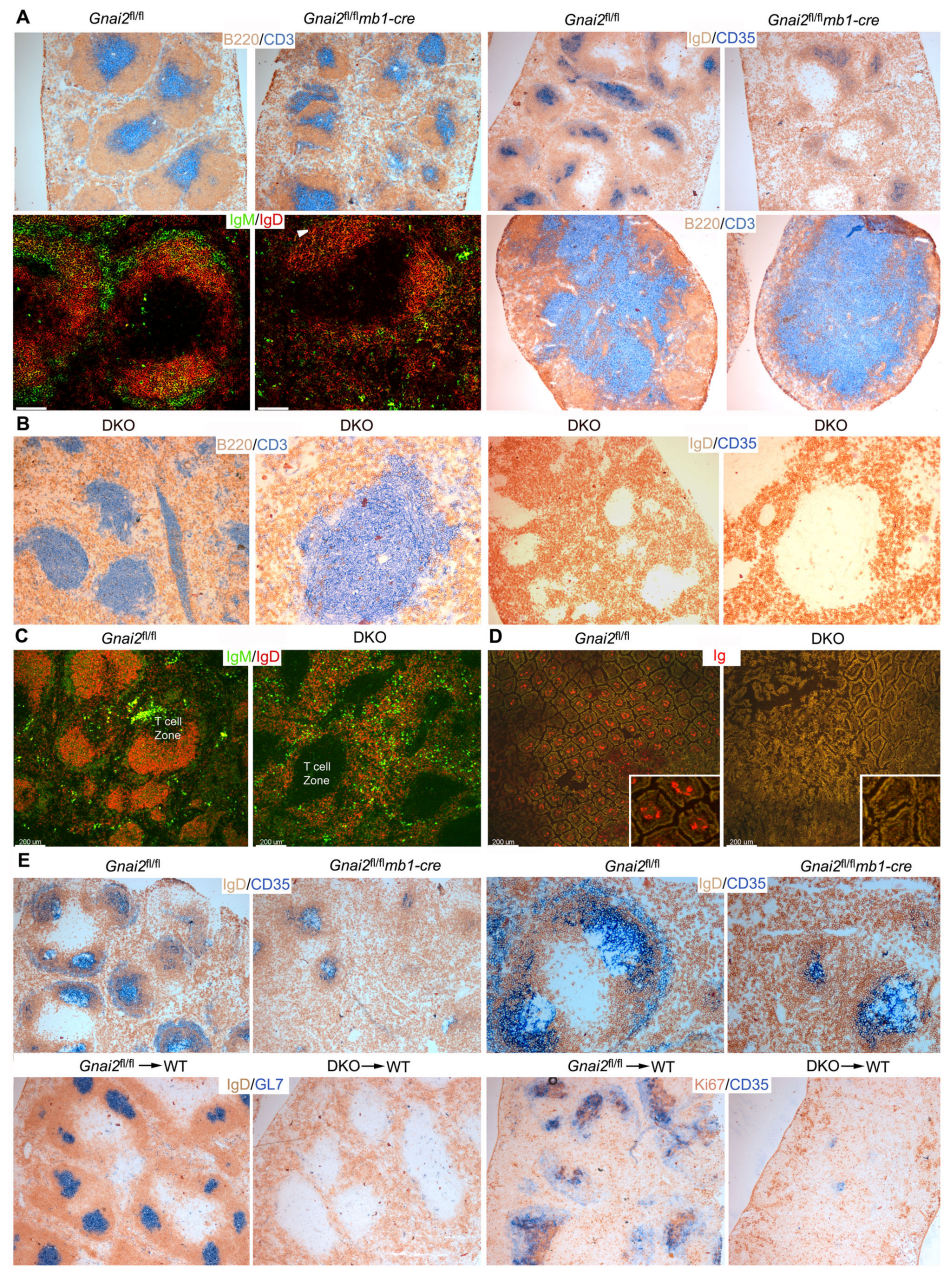
The loss of B cell compartments in the DKO mice. A. Immunohistochemical and immunofluorescent analysis of slides prepared from control and *Gnai2* ^fl/fl^ *mb1-cre* mice. Spleen and mesenteric lymph node (bottom right) sections were immunostained as indicated. Immunohistochemistry are 4X images and the immunofluorescent are 10X. Arrowhead delineates marginal zone region (MZ). Scale bars are 50 µm. B. Immunohistochemical analysis of spleen sections prepared from DKO mice. The sections were processed as indicated. Adjacent images 4X and 10X. C. Confocal microscopy using control and DKO mouse spleens immunostained for IgM and IgD. Scale bars are 200 µm. D. Confocal microscopy using sections prepared from the ileum of control or DKO mouse immunostained for Ig. Insert electronically magnified 2.5X. Scale bars are 200 µm. E. Immunohistochemistry of spleen sections from control and *Gnai2* ^fl/fl^ *mb1-cre* mice (above) or bone marrow re-constituted mice (below) that had been immunized 9 days earlier with sheep RBCs. Sections were immunostained with the indicated antibodies. The IgD/CD35 images were collected with a 4X objective (left) and 10X (right). The images below were collected with a 4X objective. C57/BL6 mice were reconstituted with *Gnai2* ^fl/fl^ or DKO bone marrow 7 weeks prior to immunization. All results are representative of at least 4 mice of the same genotype.

Next, we examined the B cell compartments in the *Gnai2* ^fl/fl^ *mb1-cre* and DKO mice using immunohistochemistry. Compared to controls the *Gnai2* ^fl/fl^ *mb1-cre* mice spleens had smaller B cell follicles, more red pulp B cells, scarcer follicular dendritic cells (FDCs), fewer B cells in the marginal zone, but an intact marginal zone sinus (Figure 7A and data not shown). These mice also had small Peyer’s patches with a distorted architecture; LNs with small B cell zones; and a severe reduction in B220^+^ cells present in the thymus (Figure 7A, and data not shown). The DKO had much more profound abnormalities. The spleen lacked B cell follicles and FDCs, and any discernible marginal zone B cells; however, a marginal zone sinus was evident closely abutted to the T cell zone (Figure 7B, and data not shown). Numerous IgM plasma cells were scattered throughout the spleen rather than focused in the bridging channels as in the wild type mice (Figure 7C). Peyer’s patches were invisible to the naked eye; the mesenteric LNs were small and lacked B cells (data not shown); and the small intestine lacked plasma cells (Figure 7D). When immunized the *Gnai2* ^fl/fl^ *mb1-cre* mice developed small poorly zoned GCs in their spleens while the DKO mice failed to form any splenic GCs (Figure 7E). These results show that the loss of Gα_i2_ in B cells severely impacts the normal architecture of the spleen, LNs, and Peyer’s patches while the loss of both Gα_i2_ and Gα_i3_ totally disrupts the organization of the B cell compartments in the spleen, LNs, and at mucosal sites.

### DKO mice exhibit a hyper IgM-like syndrome while the Gnai2 ^fl/fl^mb1-cre mice have a skewed response to NP-Ficoll immunization

**Figure 8:**
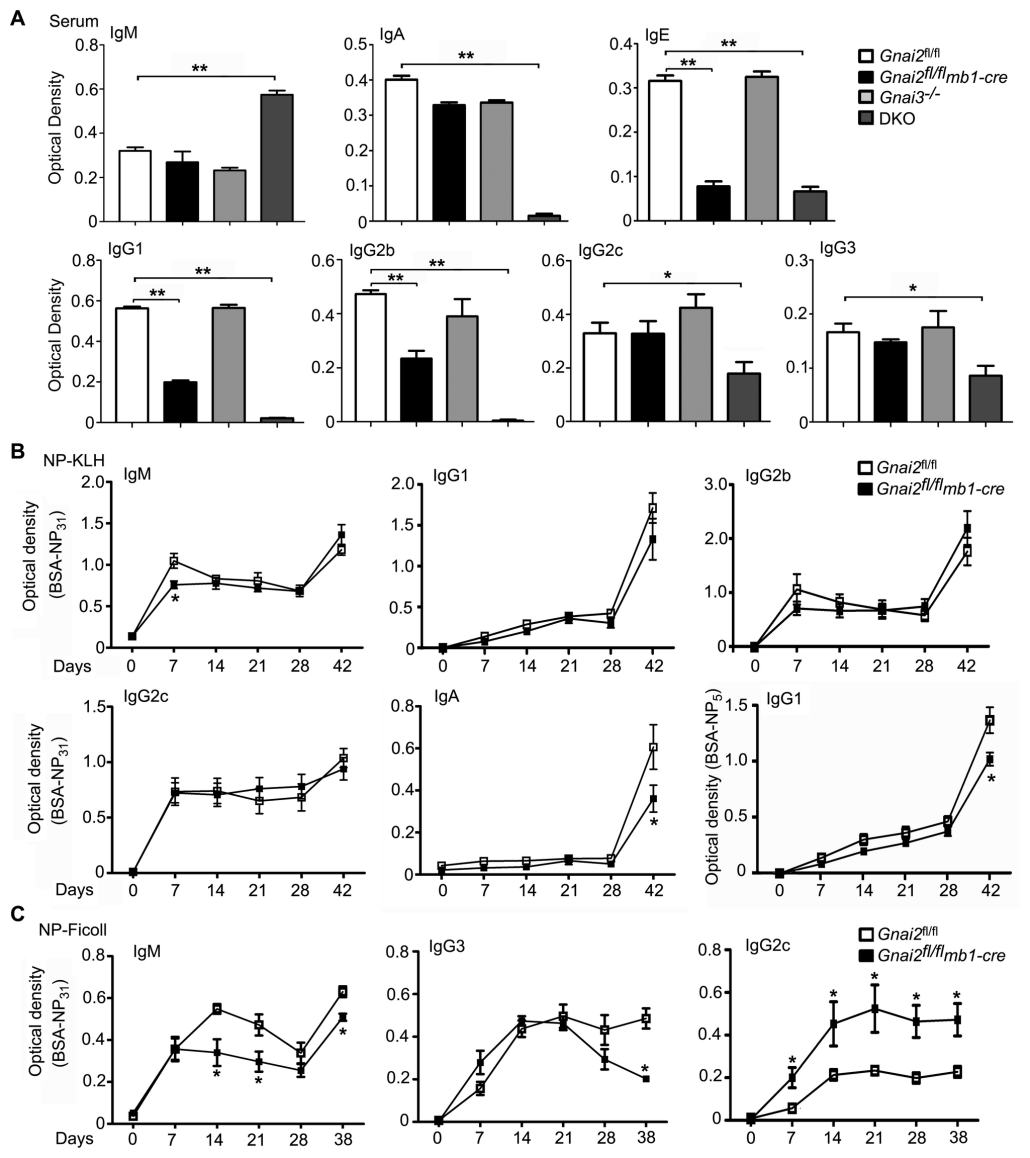
Hyper-IgM like syndrome in the DKO mice and impaired antibody responses in the G*nai2* ^fl/fl^ *mb1-cre* mice. A. ELISA assay to measure immunoglobulin isotypes in the sera of control and mutant mice. Sera analyzed were from 6 mice for each genotype. Data is shown as mean +/- SEM. Statistics from the analysis of the data using *t* test. B. ELISA assay measuring specific antibody in the serum of NP-KLH immunized control and *Gnai2* ^fl/fl^ *mb1-cre* mice. Four mice of each genotype were immunized and serum collected at the indicated time points. Mice were boosted at day 35. Results from individual time points were compared and statistical significance determined by *t* test. Data shown as mean +/- SEM. C. ELISA assay measuring specific antibody in the serum of NP-Ficoll immunized control and *Gnai2* ^fl/fl^ *mb1-cre* mice. Four mice of each genotype were immunized and boosted at day 35. Sera collected at the indicated time points. ELISA results from individual time points were compared and statistical significance determined by *t* test. Data shown as mean +/- SEM. ** p<0.001, * p<0.05.

Finally, we examined immunoglobulin levels in the serum of the different mice and tested the ability of the *Gnai2* ^fl/fl^ *mb1-cre* mice to mount an antibody response to NP-KLH and NP-Ficoll. The Gnai3^-/-^ mice had serum immunoglobulin levels similar to that of wild type mice (Figure 8A). While the *Gnai2* ^fl/fl^ *mb1-cre* mice had normal levels of IgM, IgA, IgG2c, and IgG3; IgE, IgG1, and IgG2b levels were depressed (Figure 8A). The DKO mice had a hyper IgM-like syndrome with elevated levels of IgM along with severely reduced levels of IgA, IgE, IgG1, and IgG2b. The serum IgG2c and IgG3 levels were also reduced although to a lesser amount (Figure 8A). When challenged with NP-KLH the *Gnai2* ^fl/fl^ *mb1-cre* mice displayed a relatively normal response with only a slight delay in their specific IgM response and a reduced IgA response following boost (Figure 8B). However, consistent with a modest reduction in the affinity maturation of their NP response an ELISA assay performed with a lower hapten-derivatized version of NP-BSA revealed a poorer IgG1 response following NP-KLH boost (Figure 8B, last panel). Consistent with their reduced marginal zone B cell compartment NP-Ficoll immunization of the *Gnai2* ^fl/fl^ *mb1-cre* mice showed a reduction in the specific IgM response with an increased IgG2c response (Figure 8C). These data indicate that the loss of Gα_i2_ in B cells impairs the GC response and distorts marginal B cell response. Despite the near absence of lamina propria plasma cells, B1 cells, and marginal zone B cells the DKO mice maintained an elevated serum level of IgM.

## Discussion

Here we show an absolute dependence upon B cell expression of Gα_i2_ and Gα_i3_ for the normal architecture of B-lymphocyte compartments and for B cell chemoattractant responses. The loss of Gα_i2_ in B cells reduces the B cell compartments sizes in LNs; in white pulp and marginal zone regions of the spleen; and at mucosal sites. The loss of Gα_i3_ is largely compensated for by Gα_i2_; however, minor abnormalities exist in the *Gnai3* deficient mice. These may arise from loss of Gα_i3_ in B cells, but also from its loss in other cell types. The loss of both Gα_i2_ and Gα_i3_ in B cells profoundly affects B cell compartments. The one exception is the bone marrow, where B cell development proceeds surprisingly normally. However, once B cells lacking Gα_i2_ and Gα_i3_ leave the bone marrow and enter the blood their only destiny is to reside in the spleen, where follicular B cell maturation proceeds despite the lack of B cell follicles, although marginal zone B cells development is aborted. However, there is a significant reduction in splenic B cell numbers and a partial differentiation block at the T0-T1 transition. The loss of B cell compartment organization is accompanied by a near complete absence of FDCs and antigen-induced GC formation. The absence of Gα_i2_ in B cells reduces chemoattractant receptor signaling by 70-90%, loss of Gα_i3_ has little observable effect, and lack of both G-proteins eliminates chemoattractant responses. Other Gα proteins cannot compensate for the absence of Gα_i2_ and Gα_i3_. Their loss also impacted the expression of several chemoattractant receptors and other homing molecules, and affected B cell antigen receptor signaling. While many of the abnormalities noted in the DKO mice can be ascribed to loss of signaling through known chemoattractant receptors as discussed below several cannot.

In these studies we deleted *Gnai2* using *mb1-cre*. The deletion should begin during early B cell development as *mb1* expression begins in Fr. A cells. Indicating that Gα_i2_ expression was declining in these cells, *Gnai2* ^fl/fl^ *mb1-cre* Fr. A cells exhibited a slight decrease in their responsiveness to CXCL12. Once the progenitors had entered Fr. B they no longer responded. CXCL12 signaling has been attributed a role in the development of very early B cell progenitors, prior to their expression of *mb1* [36]. A phenotype not recapitulated in our mice likely due to the timing of *cre* expression. The lack of CXCL12/CXCR4 signaling axis also results in an early release of bone marrow B cell progenitors, while deficiency of *S1pr1* or *Cnr2* interferes with their release [12,13]. The loss of Gα_i2_ and Gα_i3_ resulted in an amalgam of the previous GPCR knockout phenotypes with evidence of immature B cell retention in the bone marrow witnessed by the increase in Fr. D cells, but also evidence of premature release as Fr. D and T0 B cells were increased in the blood. As it has been proposed that these immature B cells can re-enter into the bone marrow via a CXCR4 dependent mechanism [37], their elevated numbers in the blood of the DKO mice may also arise from a failure of this recruitment. The reduction in the numbers of Fr. F cells, mature re-circulating B cells, in the DKO mice bone marrow is likely explained by the same mechanism as CXCR4 signaling cannot recruit them into peri-sinusoidal niches [38]. Yet B cell development and B cell egress from the bone marrow was less impacted by the B cell specific loss of Gα_i2_ and Gα_i3_ than were other B cell compartments.

For example, the DKO mice had a severe disruption of splenic B cell differentiation and splenic architecture. Similar to Ebi2^-/-^Cxcr5^-/-^Ccr7^-/-^ deficient mice [39], the lack of Gα_i2_ and Gα_i3_ in B cells led to an absence of B cell follicles in the spleen. However, in contrast to these mice, the DKO mice had intact T cell zones as T cell CCR7 signaling was not affected. The DKO mice lacked splenic marginal zone B cells, which were present in the Ebi2^-/-^Cxcr5^-/-^Ccr7^-/-^ deficient mice. As argued previously an unknown GPCR likely supports proper marginal zone B cell development. Candidate GPCRs based on their expression patterns would include Gpr156, Gpr18, or Gpr43. Surprisingly, the disorganized lymphoid architecture in the Ebi2^-/-^Cxcr5^-/-^Ccr7^-/-^ deficient mice did not affect splenic B cell differentiation, nor apparently, splenic B cell numbers [39]. This contrasts with the mice lacking Gα_i2_ and Gα_i3_ in their B cells, which had a 50-60% reduction in splenic B cells and a significant block in the generation of T1 B cells. These observations suggest that a GPCR-mediated signal other than that delivered by CXCR5, CCR7, or Ebi2 supports splenic B cell differentiation. Our results also contrast with mice whose B cells lack Rac1 and Rac2 expression [37]. In those mice, B cell development in the spleen arrested at the T0 stage. It was proposed that the loss of Rac1 and Rac2 prevented further splenic B cell development because transitional B cells could not migrate into the white pulp to receive survival signals. However, the DKO B cells cannot enter the while pulp, yet splenic B cell differentiation generated cells of a mature B cell phenotype. The previous analysis of the Ebi2^-/-^Cxcr5^-/-^Ccr7^-/-^ mice also indicates that that migration into the white pulp is not needed for splenic B cell survival [39]. The nature of the B cell differentiation defect in the absence of Gα_i2_ and Gα_i3_ signaling in B cells needs further investigation. Since normal immature B cells differentiate to T1 B cells regardless of BAFF-R signaling [40], an effect of Gα_i_ signaling on BAFF/BAFF-R axis cannot explain the decrease of T1 cells in the DKO mice. A reduction in tonic BCR signaling and BCR expression reduced the differentiation of immature B cells into transitional and mature B cells [41]. As the DKO B cells exhibited a reduced intracellular calcium response to BCR crosslinking this may help explain the differentiation defect and, if this is the case, would link Gα_i_ to the BCR signaling pathway.

Despite the presence of an organized T cell compartment no organized B cell structures were present nor were FDCs, yet a marginal zone sinus was present closely abutted to the T cell zone. In the DKO mice the inability of CXCL13 to recruit B cells to developing follicles where their production of lymphotoxin α_1_β_2_ and tumor necrosis factor helps drive FDC development likely explains the lack of FDCs [42,43]. In contrast to B cell deficient mice, which have disturbed splenic T cell zones and reduced number of splenic T cells [44], the DKO mice had relatively normal appearing T cell zones and normal numbers of CD4 and CD8 T cells. This indicates that B cell chemoattractant receptor signaling is dispensable for B cells to support the architecture of the T cell zone in the spleen and for the development of the marginal zone sinus. In addition, splenic GC formation was impaired in the *Gnai2* ^fl/fl^ *mb1-cre* mice and absent in the DKO mice. The lack of B cell expression of Gα_i2_ reduced the numbers and sizes of splenic GCs, and those GCs that formed lacked obvious light and dark zones. These abnormalities can be attributed to defective Ebi2, CCR7, CXCR4 and CXCR5 signaling in the B cells [45,46].

Mature B cells were present in the blood yet only a rare B cell resided in peripheral LNs. Blood B cells use CCR7 and to a lesser degree CXCR4 signaling to adhere to and to cross high endothelium venules (HEVs) in peripheral LNs [9,16]. When adoptively transferred into wild type mice the DKO B cells rarely cell entered LNs. Those few cells that did exhibited little or no motility, consistent with previous studies implicating chemokines in driving B cell motility in LNs [47]. Although the LNs of the DKO mice largely lacked B cells, LN development was unimpaired in these mice. This contrasts with the CXCR5/CCR7 DKO and Gnai2^-/-^ mice, which are missing or often missing their peripheral LNs [8,48].

In the DKO mice mesenteric LNs essentially lacked B cells, Peyer’s patches were invisible, and the lamia propria was devoid of plasma cells. Early Peyer’s patch development should be intact in these mice as B cells are unnecessary for Peyer’s patch formation [49]. Peyer’s patch inducer cells play a key role in the formation of the follicular zone by stimulating B cell accumulation and the formation of FDCs [49]. In the DKO mice this recruitment does not occur and B cell zones will never develop. Since FDCs and B cells help maintain the organization of the Peyer’s patches [50], over time the Peyer’s patch apparently regresses. No Peyer’s patches were visible 2 months after birth or 2 months after reconstitution of wild type mice with DKO bone marrow. The DKO mice demonstrate a near total reliance on B cell chemokine receptor signaling for B cells and plasma cells to populate the lamina propria and other mucosal sites.

Consistent with this role IgA serum levels were markedly depressed in the DKO mice. Indeed, the serum immunoglobulin profile observed resembled that of hyper IgM syndrome with elevated IgM and depressed levels of other Ig isotypes. The hyper IgM syndromes are a group of rare inherited immune deficiency disorders that are characterized by impairment of immunoglobulin isotype switching. Known causes include mutations in *CD40*, *CD40L*, *Aidca*, and *Ung*; and defects in class switch recombination [51,52]. Interactions between CD40 and CD40L are needed for B cell differentiation and subsequent isotype switching. Mutations in individual chemokine or chemokine receptors have not been associated with a hyper IgM-like syndrome. Even the loss of both CXCR5 and CCR7 does not significantly reduce serum IgG and IgA levels [53]. In the DKO mice splenic follicular B cells must be the source of the serum IgM as these mice essentially lack other B cells. Since the DKO mice have no intrinsic defects in Ig class switching, a failure of normal cellular interactions may explain the sharp reduction in serum IgG. By comparison the serum immunoglobulin profile in the *Gnai2* ^fl/fl^ *mb1-cre* mice was relatively normal with only modest reductions in IgE, IgG1, and IgG2b. Despite the defect in GC formation and zoning in these mice the response to NP-KLH did not differ significantly from that of control mice with the exception of a retarded specific IgG1 response and slightly reduced affinity of the secondary response. These mice had a more defective antibody response to NP-Ficoll perhaps as a consequence of fewer marginal zone and B1 B cells. However, while the mice had a subpar IgM and IgG3 response they had a more robust IgG2c response. This may be a compensatory response due to the reduced marginal zone and B1 cells as NP-Ficoll can trigger follicular B cells to make IgG2c [54].

In conclusion B cell chemoattractant signaling is highly dependent upon Gα_i2_, but absolutely dependent upon Gα_i2_ and Gα_i3_. In their absence the usual B cell compartments in the spleen, LNs, and at mucosal sites no longer exist. GC formation fails and a hyper IgM-like syndrome ensues. However, eliminating Gα_i2_ and Gα_i3_ at the pro-B cell stage only modestly affects B cell development and bone marrow egress. Splenic follicular B cell development takes place although less efficiently. In contrast, marginal zone B cell development is crippled suggesting a role for an unidentified GPCR. B1 cells are severely reduced in the peritoneum presumably due to a failure of CXCR5 signaling. Further studies of the DKO mice as well as Gnai3^-/-^ mice in which the cre-mediated deletion of *Gnai2* is targeted to specific B cell stages or other hematopoietic cell types should provide new insights into chemoattractant receptor signaling in these cells.
